# Empowering Psychiatric Trainees: Enhancing Portfolio Competence Through the Café of Royal College of Psychiatrists (CoRP) Quality Improvement Project

**DOI:** 10.1192/bjo.2024.389

**Published:** 2024-08-01

**Authors:** Jiann Lin Loo, Praveen Kumar, Kok Keong Leong, Justina Akinlua, Sioned Griffiths

**Affiliations:** 1 Betsi Cadwaladr University Health Board, Wrexham, United Kingdom; 2 New Craig's Psychiatric Hospital, Inverness, United Kingdom; 3 Aneurin Bevan University Health Board, Blaenau Gwent, United Kingdom; 4 Betsi Cadwaladr University Health Board, Llanfairfechan, United Kingdom; 5 Betsi cadwaladr University Health Board, Bangor, United Kingdom

## Abstract

**Aims:**

The Quality Improvement Project (QIP) for the Café of Royal College of Psychiatrists (RCPsych) Portfolio (CoRP) was initiated to address the challenges faced by UK postgraduate psychiatric trainees in utilizing the RCPsych Portfolio effectively. The primary objective of this project is to enhance trainees' confidence and competence in using the portfolio. Additionally, CoRP aims to establish a robust, sustainable ecosystem of peer coaching and mentorship to support continuous learning and development among trainees.

**Methods:**

The CoRP employs a unique, multi-faceted approach, leveraging a scalable coaching and mentoring model. Firstly, the program focuses on increasing its visibility among trainee groups through targeted communication and marketing efforts. Secondly, CoRP provides on-demand sessions to cater to the varied schedules and job plans of trainees, offering flexibility and accessibility. The sessions offer a mix of coaching, mentorship, and guidance, tailored to the specific needs and learning styles of each trainee. Furthermore, the project fosters an environment where trainees can learn from peers and experienced professionals, enhancing the learning experience and promoting a culture of collaborative learning.

**Results:**

The implementation of the CoRP has led to significant improvements in trainees' confidence in using the RCPsych Portfolio. This outcome is evidenced by the data collected from pre- and post-session surveys, which show a marked increase in trainees' self-reported confidence levels. The project has successfully conducted a series of sessions that focus on various aspects of portfolio management and learning. These sessions have been well-received, with positive feedback from participants indicating that the program meets its intended objectives. However, the project acknowledges the need for long-term data to understand its impact on the Annual Review of Competency Progression (ARCP) outcomes and to assess its sustainability over time.

**Conclusion:**

The CoRP has demonstrated immediate, positive effects in enhancing the skills of psychiatric trainees in using the portfolio. Its strengths lie in the scalability of the model and the incorporation of coaching and mentorship principles, which have proven effective in addressing the needs of trainees. However, the project recognizes that further evaluation is needed to establish a clear correlation between improved portfolio skills and ARCP outcomes. To this end, future plans include the continuous expansion and repetition of the program every six months to accommodate new trainees. Additionally, ongoing evaluation will be conducted to measure the program's long-term effectiveness and sustainability. This will ensure that CoRP continues to evolve and adapt to the changing needs of psychiatric trainees, ultimately contributing to their professional development and success in their field.

